# Video-Based versus On-Site Neonatal Pain Assessment in Neonatal Intensive Care Units: The Impact of Video-Based Neonatal Pain Assessment in Real-World Scenario on Pain Diagnosis and Its Artificial Intelligence Application

**DOI:** 10.3390/diagnostics13162661

**Published:** 2023-08-12

**Authors:** Xiaofei Chen, Huaiyu Zhu, Linli Mei, Qi Shu, Xiaoying Cheng, Feixiang Luo, Yisheng Zhao, Shuohui Chen, Yun Pan

**Affiliations:** 1 Gastroenterology Department, The Children’s Hospital, Zhejiang University School of Medicine, National Clinical Research Center for Child Health, Hangzhou 310052, China; hzxiao0914@163.com; 2 College of Information Science and Electronic Engineering, Zhejiang University, Hangzhou 310027, China; zhuhuaiyu@zju.edu.cn (H.Z.); zhaoys@zju.edu.cn (Y.Z.); 3 Administration Department of Nosocomial Infection, The Children’s Hospital, Zhejiang University School of Medicine, National Clinical Research Center for Child Health, Hangzhou 310052, China; 22018565@zju.edu.cn (L.M.); 22118788@zju.edu.cn (Q.S.); 4 Quality Improvement Office, The Children’s Hospital, Zhejiang University School of Medicine, National Clinical Research Center for Child Health, Hangzhou 310052, China; cxynicu@163.com; 5 Neonatal Intensive Care Unit, The Children’s Hospital, Zhejiang University School of Medicine, National Clinical Research Center for Child Health, Hangzhou 310052, China; luofeixiang@zju.edu.cn

**Keywords:** neonatal pain assessment, inter-rater variability, neonatal intensive care units, neonatal nursing, pain management

## Abstract

Background: Neonatal pain assessment (NPA) represents a huge global problem of essential importance, as a timely and accurate assessment of neonatal pain is indispensable for implementing pain management. Purpose: To investigate the consistency of pain scores derived through video-based NPA (VB-NPA) and on-site NPA (OS-NPA), providing the scientific foundation and feasibility of adopting VB-NPA results in a real-world scenario as the gold standard for neonatal pain in clinical studies and labels for artificial intelligence (AI)-based NPA (AI-NPA) applications. Setting: A total of 598 neonates were recruited from a pediatric hospital in China. Methods: This observational study recorded 598 neonates who underwent one of 10 painful procedures, including arterial blood sampling, heel blood sampling, fingertip blood sampling, intravenous injection, subcutaneous injection, peripheral intravenous cannulation, nasopharyngeal suctioning, retention enema, adhesive removal, and wound dressing. Two experienced nurses performed OS-NPA and VB-NPA at a 10-day interval through double-blind scoring using the Neonatal Infant Pain Scale to evaluate the pain level of the neonates. Intra-rater and inter-rater reliability were calculated and analyzed, and a paired samples *t*-test was used to explore the bias and consistency of the assessors’ pain scores derived through OS-NPA and VB-NPA. The impact of different label sources was evaluated using three state-of-the-art AI methods trained with labels given by OS-NPA and VB-NPA, respectively. Results: The intra-rater reliability of the same assessor was 0.976–0.983 across different times, as measured by the intraclass correlation coefficient. The inter-rater reliability was 0.983 for single measures and 0.992 for average measures. No significant differences were observed between the OS-NPA scores and the assessment of an independent VB-NPA assessor. The different label sources only caused a limited accuracy loss of 0.022–0.044 for the three AI methods. Conclusion: VB-NPA in a real-world scenario is an effective way to assess neonatal pain due to its high intra-rater and inter-rater reliability compared to OS-NPA and could be used for the labeling of large-scale NPA video databases for clinical studies and AI training.

## 1. Introduction

Procedural pain in newborns represents a growing concern due to the increasing number of invasive procedures these patients undergo while receiving healthcare. Advances in neonatal care have promoted the survival of premature and sick infants; however, this has come at the cost of repeated episodes of acute and/or prolonged pain [1,2,3]. In neonatal intensive care units (NICUs), newborns are often subjected to injections and blood draws without the use of analgesia medication [4], which results in higher sensitivity to pain compared to older infants, children, and adults [5]. Pain can cause clinical instability, such as changes in cardiac and respiratory frequencies, and can even lead to complications, such as interventricular hemorrhages [6,7]. In order to ensure proper pain management, timely and accurate neonatal pain assessment (NPA) is of essential importance [8]. However, a cross-sectional study found that only 32.5% of pain records adopted pharmacological or non-pharmacological intervention for pain relief [9].

As newborns cannot self-report, caregivers must assess their pain by observing specific behavioral and physiological signs. This is usually conducted by using pediatric scales. Currently, there are more than 40 scales designed for this purpose; however, such an assessment approach is highly biased and is affected by several idiosyncratic factors, such as the observer’s cognitive bias, identity, background, and culture, as well as gender, resulting in inconsistent assessment and treatment of pain [10]. In addition, current pain management of newborns in the NICU is manually performed, being subjective and discontinuous, with NICU nurses treating neonates with pain management plans based on intermittent, subjective ratings with poor inter-rater agreement [11]. Furthermore, the current practice for assessing infants’ pain is time-consuming and requires many trained and professional laborers.

Pain is recognized as the fifth vital sign that should be monitored in NICUs [12]. Despite the growing body of literature on pain assessment and clinical practice guidelines that emphasize the importance of pediatric pain management, many pediatric patients still receive inadequate pain treatment. This is mainly due to the time and effort needed to evaluate pain, a lack of pain experts, and inadequate education on pain management among pediatric trainees. Additionally, cultural or personal beliefs such as negative attitudes towards pain treatment, the belief that pain builds character, and fear of adverse effects of pain medications can lead to improper pain management [13]. Despite numerous guidelines and standards requiring the use of standardized pain assessment tools in clinical practice, there is still poor compliance, posing a serious global issue [11,13].

To solve the above problems, both clinical NPA studies and new NPA technologies, such as artificial intelligence (AI)-based NPA (AI-NPA), should be developed to improve the quality and efficiency of current NPA for effective neonatal pain management. Those studies generally require large-scale neonatal pain data, e.g., neonatal images, videos, and physiological signals collected in pain, with precise pain diagnosis results for clinical statistical analysis or AI training, where neonatal pain videos with pain scores given by a consultation group of nursing experts using a pain scale is a common and feasible data form [11]. However, considering the real-world NICU scenario, it is difficult to carry out such labeling work with multiple experienced nurses on-site for large-scale data.

As the video-based NPA (VB-NPA) protocol could facilitate remote or after-the-event pain diagnosis by experts, it is widely used in clinical NPA and AI-NPA research as an equivalent alternative to the gold standard on-site NPA (OS-NPA) and has been proven feasible for ideal neonatal pain video data captured in controlled conditions; that is, intentional controls during the data collection phase or manual data selections at the pre-processing stage to ensure complete neonatal pain responses are captured with a correct perspective in neonatal pain videos. Yet neonatal pain videos captured in a real-world scenario could contain various disturbances, such as facial occlusion, pose variation, body occlusion, and movement interference from others. These real-world noises would cause information loss in videos, which might be crucial to NPA and further make VB-NPA lose its advantages, even its equivalence with OS-NPA.

In this paper, we investigated whether VB-NPA with neonatal pain videos captured in a real-world NICU scenario is with the consistency of OS-NPA and could be used for AI-NPA applications. A total of 598 neonates hospitalized in the NICU for more than 3 days and scheduled for a procedural pain procedure were randomly selected and included in the study. Both the OS-NPA and VB-NPA after 10 days were performed by two nurses in the form of a pain score and pain grade with reference to the Neonatal Infant Pain Scale (NIPS) [14]. Using the NIPS pain score of the OS-NPA as the golden standard, the result showed a high intraclass correlation coefficient (ICC) and inter-rater reliability for both single and average measures between the VB-NPA, with neonatal pain videos captured in a real-world NICU scenario, and OS-NPA, with a highly significant correlation (*p* < 0.001).

Compared with the on-site evaluation, the accuracy of the NIPS pain grade given by the VB-NPA was 96.98%, and the agreement between the two groups was compared, with a kappa value of 0.926 (*p* < 0.001), thus indicating that VB-NPA with neonatal pain videos captured in a real-world NICU scenario could cause inaccuracies in partial scoring due to the information loss in the videos, yet it was still not inferior to OS-NPA. Meanwhile, the test results of three state-of-the-art AI-NPA methods only showed an accuracy loss of 0.022–0.044, which was caused by the VB-NPA labels, indicating that there was just a limited impact of VB-NPA with neonatal pain videos captured in a real-world NICU scenario to AI-NPA. Therefore, VB-NPA in a real-world NICU scenario is an effective way to assess neonatal pain due to its high intra-rater and inter-rater reliability compared to OS-NPA and could be used for the labeling of large-scale NPA video databases for clinical studies and AI training.

## 2. Methods

### 2.1. Setting and Participants

**Figure 1:**
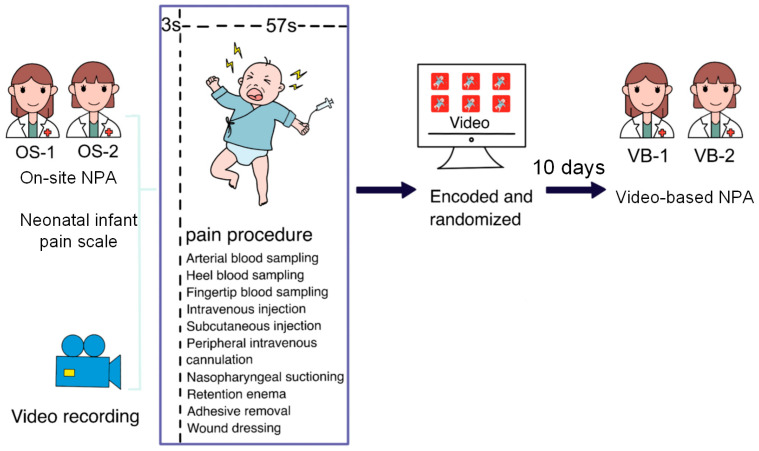
The study design of the neonatal pain assessment (NPA).

This study was conducted at a tertiary class of a children’s hospital in eastern China between 1 December 2021 and 30 May 2022. It was approved by the ethics committee of the Children’s Hospital of Zhejiang University School of Medicine (2018-IRB-051) on 31 July 2018, and parental informed consent was obtained. The study flowchart is shown in Figure 1.

A total of 598 neonates hospitalized in the NICU for more than 3 days and already scheduled for a procedural pain procedure were randomly selected and included in the study. All hospitalized newborns underwent standard neonatal disposal after admission, including uniform clothing changing after admission. The procedures included arterial blood sampling, heel blood sampling, fingertip blood sampling, intravenous injection, subcutaneous injection, peripheral intravenous cannulation, nasopharyngeal suctioning, retention enema, adhesive removal, and wound dressing. These procedures were characterized as painful by doctors and nurses working in pediatrics and neonatology [3]. Exclusion criteria referred to serious illnesses such as birth injury, asphyxia, shock, metabolic encephalopathy, hypoxic-ischemic encephalopathy, severe cardiopulmonary disease, and conditions associated with facial image acquisition, such as severe congenital malformations.

Two experienced nurses were assigned to quantify the pain of 10 types of procedures for newborns using the NIPS on-site, with a third nurse recording the procedure simultaneously. The double-blind scoring results of OS-NPA performed by the two nurses were recorded as OS-1 and OS-2, respectively. All recorded videos were encoded and stored in the software, and the chronological sequence of the videos was randomized using a random number table to blind the assessors. After 10 days, the same two nurses performed VB-NPA on these randomized videos, and the double-blind scoring results were recorded as VB-1 and VB-2, of which the index numbers denoted the same nurse.

### 2.2. Video Recording in Real-World Scenario

**Figure 2:**
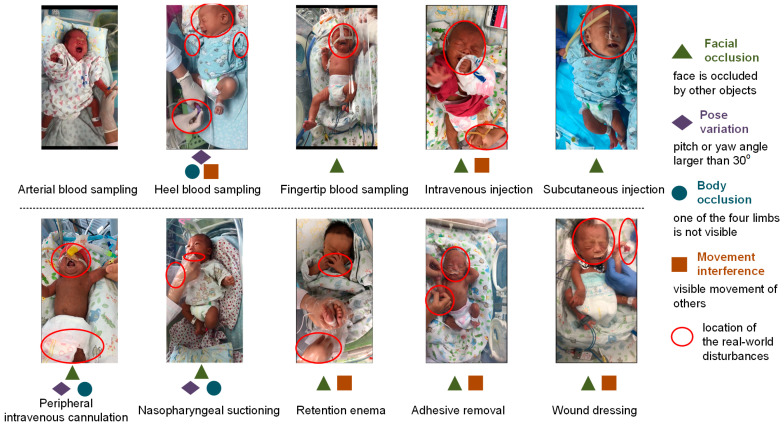
Visual representation of pain samples from neonatal pain videos captured in a real-world NICU scenario without control.

The duration of painful procedures was limited to one minute. Meanwhile, a third nurse recorded the newborn’s behavior in a 1-min video starting 3 s before the procedure. The video recordings were taken with a smartphone with automatic stabilization and a resolution of 1334 × 750 from 12 megapixels. There are no special restrictions during video shooting to guarantee recorded neonatal pain responses unaffected by occlusion, interference from other people’s movements, or extreme postures of newborns. The sample key frames of these neonatal pain videos are shown in Figure 2.

### 2.3. Pain Assessment

The NIPS was developed in the early 1990s at the Children’s Hospital of Eastern Ontario to assess six behavioral reactions to painful procedures in preterm and full-term newborns [14]. Subsequently, the NIPS was successfully adapted and validated for use in other countries [15,16]. Its total score ranged from 0 to 7 points: facial expression (0–1 point), cry (0–2 points), breathing pattern (0–1 point), the position of arms (0–1 point), the position of legs (0–1 point), and state of arousal (0–1 point), with 0 being no pain and 7 being the most intense pain. The NIPS is easily understood and applied and is a useful tool for health professionals who work with neonates exposed to painful stimuli. Previous studies demonstrated that the scale has high inter-rater reliability and internal consistency [17]. It was also validated for construct and concurrent validity, making it a valid, reliable, and practical tool. Cronbach’s alpha values of the Chinese version of the NIPS were found to be 0.97, 0.81, and 0.95 before, during, and after the heel lance, respectively [18].

### 2.4. OS-NPA and VB-NPA

In this study, we compared the consistency between the two NPA methods, i.e., OS-NPA, which involves medical staff observing the newborn’s behavior on-site, and VB-NPA, which involves medical staff observing the newborn’s behavior through a video recording. For the OS-NPA, two experienced nurses evaluated the pain scores of newborns undergoing painful procedures on-site using the NIPS. All videos captured in the real-world NICU scenario were randomized using a random number table to obfuscate the subject and timing information of the video. They were randomized and uploaded to in-hospital web-based video-rating software to ensure that the OS-NPA nurses were blinded during the VB-NPA. In order to minimize construct-irrelevant variance, VB-NPA training was conducted after the two nurses watched and assessed 5 videos, respectively, to increase the accuracy of the assessments, and after 10 days, the two OS-NPA nurses again evaluated neonatal pain using the NIPS through the recorded videos to derive their corresponding VB-NPA results.

### 2.5. Data Analysis

Intra-rater reliability was explored by comparing the NPA results under OS-NPA with the same assessor’s results under VB-NPA (OS-1 vs. VB-1 and OS-2 vs. VB-2). Inter-rater reliability was explored by comparing assessments based on video recordings (VB-1 vs. VB-2). Reliability measures were calculated using the ICC for single and average measures. To investigate whether there was a Hawk–Dove effect between the two assessors, a paired samples *t*-test was conducted to compare NIPS pain scores given between OS-1 and OS-2; VB-1 and VB-2 statistical analysis was conducted using SPSS version 26.0 (IBM, Armonk, NY, USA).

The ICC was used to evaluate the repeatability or consistency of different measurement methods or assessors to the same certain measurement results. The randomized, double-blind method was applied, considering the influence of newborns and nurses in evaluating the reproducibility of diagnostic tests. The absolute agreement can be used to measure whether different investigators provide the same absolute value. The analysis unit of single measures is the results of each investigator, which can be used to estimate the situation of an individual investigator. Average measures are the mean of the research results of multiple investigators.

Furthermore, to investigate the impact of different label sources, i.e., OS-1, OS-2, VB-1, and VB-2, on artificial intelligence (AI) methods, we implemented three state-of-the-art AI-based NPA (AI-NPA) methods [19,20,21] to analyze the performance of these methods trained by the above four label sources. Zamzmi et al. [19] used an ensemble machine-learning framework to perform AI-NPA by fusing features of facial expressions, crying sounds, body movements, and vital signs; Min et al. [20] used a CNN-LSTM scheme to extract 2D features from neonatal videos and detect discomfort of neonates automatically; and Salekin et al. [21] proposed a multimodal spatio-temporal deep learning approach to analyze visual and vocal signals of neonatal videos to perform AI-NPA.

In this paper, we applied 5-fold cross-validation based on these three methods using the 598 video data we collected. The divided training and test video data were the same for the three methods in each fold. For each fold, we trained the methods using the training video data with the label given by OS-1, OS-2, VB-1, and VB-2, respectively, and evaluated the accuracy of each method using the test video data with the label given by OS-1 and OS-2, respectively, since the current common on-site scale rating is regarded as the gold standard for neonatal pain assessment.

## 3. Results

### 3.1. Study Population

A total of 598 neonates, with a mean birth weight of 2372.0 ± 1000.8 g, were recruited from a children’s hospital in China. Among them, 252 were female, and 346 were male; 270 were born by spontaneous delivery, and 328 were delivered by cesarean section. Every newborn underwent one of the 10 kinds of the above-mentioned painful procedures. The detailed basic characteristics are listed in Table 1.

### 3.2. Intra-rater Reliability and Inter-Rater Reliability

**Figure 3:**
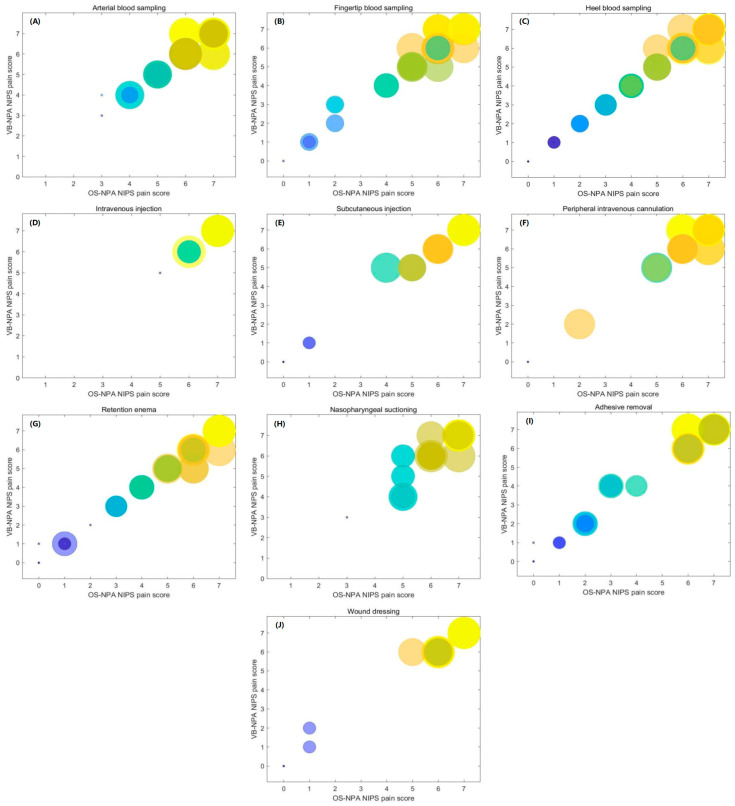
Scatter diagrams of NIPS pain scores of OS-NPA and VB-NPA: (**A**) arterial blood sampling, (**B**) fingertip blood sampling, (**C**) heel blood sampling, (**D**) intravenous injection, (**E**) subcutaneous injection, (**F**) peripheral intravenous cannulation, (**G**) retention enema, (**H**) nasopharyngeal suctioning, (**I**) adhesive removal, and (**J**) wound dressing. “NIPS” stands for the Neonatal Infant Pain Scale, “OS-NPA” refers to the on-site neonatal pain assessment, and “VB-NPA” refers to the video-based neonatal pain assessment.

The goodness of fit for the linear regression model between VB-2 and OS-1 was 0.976, as shown in Figure 3. The NIPS pain scores are represented by the size and color of the circles, with the frequency of the ratings determining the size and darkness. The larger and darker the circles are, the more frequent the ratings, indicating a high level of consistency between the two. Additionally, when comparing the results from OS-1 with VB-1, we found an intra-rater reliability of 0.976, which was strongly significant (*p* < 0.001). When comparing the results from OS-1 with VB-2, the inter-rater reliability was 0.976 for single measures (*p* < 0.001) and 0.988 for average measures (*p* < 0.001). As shown in Table 2, there was no significant difference in the means between the two assessors’ raters (*p* > 0.05). Both assessors had higher means in some types of scores, indicating a discriminative ability between the different procedures.

### 3.3. Comparison of the NIPS Pain Grades between OS-NPA and VB-NPA

According to the pain grade criteria of the NIPS, the OS-NPA showed no pain in 98 patients (16.38%), mild pain in 15 patients (2.50%), moderate pain in 36 patients (6.02%), and severe pain in 449 patients (75.08%). On the other hand, the VB-NPA showed no pain in 97 patients (16.22%), mild pain in 10 patients (1.67%), moderate pain in 42 patients (7.02%), and severe pain in 447 patients (74.74%). Compared with the on-site evaluation, the accuracy of the NIPS pain grade given by the VB-NPA was 96.98% (580/598), and the agreement between the two label sources was compared, with a kappa value of 0.926 (*p* < 0.001), as shown in Table 3.

### 3.4. Impact of Different Label Sources on AI Methods

The five-fold cross-validation accuracies for different AI-NPA methods trained and tested using different label sources, i.e., OS-1, OS-2, VB-1, and VB-2, for training with OS-1 and OS-2 for testing, are summarized in Table 4. For different AI algorithms, the highest average accuracies were all achieved when the training and testing labels were sourced from the same labeling conditions and labeling individuals (OS-1 training for the OS-1 test and OS-2 training for the OS-2 test), followed by the same labeling conditions and different labeling individuals (OS-1 training for the OS-2 test and OS-2 training for the OS-1 test), followed by different labeling conditions and the same labeling individuals (VB-1 training for the OS-1 test and VB-2 training for the OS-2 test), and the lowest accuracies were reached when the labeling conditions and labeling individuals were both different (VB-1 training for the OS-2 test and VB-2 training for the OS-1 test). However, considering the standard deviations of the accuracies for the five-fold cross-validation, the difference in the accuracies for the AI methods with different label sources was relatively small, with an accuracy loss of 0.022–0.044. Meanwhile, this phenomenon could be described as a domain migration problem in artificial intelligence and optimization based on mature domain migration methods. Therefore, we believed that the impact of different label sources on the AI methods was still limited in this study.

## 4. Discussion

### 4.1. High Evaluation Consistency of the VB-NPA

The above findings demonstrated that NPA could be accurately and reliably performed based on videos captured in a real-world NICU scenario, thus greatly advancing NICU pain management. This has important implications for the direct observation of neonatal care, as it could provide a more precise way to assess and manage pain in newborns. Pain assessment in newborns is often challenging due to their inability to communicate the discomfort, which can eventually result in inadequate pain management and serious consequences for the infant’s health and well-being. Using the NIPS pain score of the OS-NPA as the golden standard, the ICC value of OS-1 and VB-2 was 0.976, with a highly significant correlation (*p* < 0.001). The inter-rater reliability was 0.983 for the single measures (*p* < 0.001) and 0.992 for the average measures (*p* < 0.001). The same result was seen between OS-2 and VB-1. The small difference between the two groups indicated that the NIPS is suitable for repeated measurements, consistent with previous studies [14,15]. The previous studies generally compared the differences between different assessors in the same clinical scenario. In this study, we still found a high consistency by comparing the OS-NPA score and the VB-NPA score, which indicated that the results obtained by the two NPA methods are equivalent, thus making it possible to use NIPS pain scores derived by VB-NPA with neonatal pain videos captured in a real-world NICU scenario in the future.

### 4.2. VB-NPA for NIPS Pain Grades

We included 598 children and 10 different procedures consistently evaluated as painful in the clinic. The results in Table 3 show that compared with the on-site evaluation, the accuracy of the NIPS pain grade given by the VB-NPA with neonatal pain videos captured in a real-world NICU scenario was 96.98%, and the agreement between the two groups was compared, with a kappa value of 0.926 (*p* < 0.001), thus indicating that, although the accuracy of VB-NPA was affected by real-world noises in the videos, it was not inferior to OS-NPA. Previous studies have shown that compared to OS-NPA, VB-NPA could significantly reduce the time spent on pain evaluation [22,23,24,25]. Meanwhile, with the advances in technology and operating equipment, it is easier to video-record painful procedures. The administrator staff can then use these recordings to review the pain level by observing the painful procedure video remotely for in-hospital nursing quality control purposes.

In addition, VB-NPA can reduce the stressful surroundings of a clinical setting, the contextual noise, and other elements that could shift the focus of the trainees from the rating. There has been an increasing interest in using machine-learning methods for understanding human behavioral responses to pain based on the analysis of facial expressions [26,27], crying sounds [28], and body movement. Several automated methods have been introduced to automatically assess infants’ pain based on behavioral or physiological pain indicators analysis. By using AI-NPA, the nursing staff can also use these recordings to judge the pain level by observing the painful procedure video in the nurse station and taking timely intervention measures, which could greatly reduce the bedside observation time and improve work efficiency. We have already developed an artificial intelligence-based NPA (AI-NPA) tool in the early stage for 232 newborns during blood sampling in neonatal wards; the accuracies of the NIPS pain score and pain grade given by the automated NPA system were 88.79% and 95.25% [24].

### 4.3. VB-NPA for AI-NPA

In the clinical environment, one of the benefits of VB-NPA compared to OS-NPA is the possibility of using blinded assessments to reduce assessor bias. Various factors can affect the reliability and accuracy of the video rating of newborn pain assessment, such as video quality, shooting distance, shooting angle, shooting time, and the personal characteristics of the assessor [11]. While the personal factors associated with previous pain assessment experiences and the personality of the assessor cannot be removed with the blinding of VB-NPA, recording the procedure opens up the possibility of allowing multiple assessors to evaluate the same procedure to ensure the accuracy of the VB-NPA. Meanwhile, to reduce the risk of inaccurate scoring, we assigned two nurses to perform the OS-NPA in this study to avoid assessor bias, as one nurse may tend to score more strictly than others. After 10 days, the VB-NPA was conducted, allowing the nurses to forget the results of the OS-NPA and avoid any interference. These measures made the VB-NPA as accurate as possible, resulting in more reliable results. All video data were included in our pain identification database, providing the possibility of establishing a neonatal pain identification database for AI-NPA in the future.

However, considering the inaccuracy and uncertainty of data labels, which are inevitable when labels are conceptual entities and manually annotated [29] like in the NIPS, the results of this study indicate a high consistency between VB-NPA and OS-NPA, and we believe that in OS-NPA, experts can adjust their own observation perspectives appropriately and selectively evaluate whether the pain representations in the scale exist in individual newborns. While VB-NPA can be performed through multiple replays, the established shooting perspective cannot be changed after data collection, making it difficult for the experts to evaluate certain scale items. At the same time, we could see that the label sources of different labeling conditions and individuals would still introduce cross-domain problems in AI analysis, resulting in the loss of algorithm performance. Therefore, we believe that further studies for improving the accuracy of current VB-NPA to achieve a higher consistency with OS-NPA is still necessary.

### 4.4. Strengths and Limitations

The strength of this study is that it shows the real-world experience of a tertiary NICU, where the strain of everyday duties and work overload can sometimes lead to the omission of pain assessment. As a result, VB-NPA could provide a more reliable and accurate way to assess and manage pain in newborns, which could have important implications for the direct observation of neonatal care. Furthermore, it could reduce the burden on healthcare professionals, as it provides a more efficient way to assess and manage pain in newborns.

This study has some limitations. The recorded operation videos are limited by the environment, personnel, and shooting angle. Additionally, other pain operations in clinical practice were not recorded due to their low operating frequency. In the future, we hope to increase the sample size and expand the pain-causing operation database.

## 5. Conclusions

The accurate assessment of pain in the NICU is essential due to the high prevalence of painful experiences. Our results showed that the video-based assessment of neonatal pain could be reliably used, as confirmed by the high intra-rater and inter-rater reliability between direct observation and the video-based assessment, as well as the AI method-based performance evaluation, even with various disturbances in real-world NICUs. These results suggest that video-based assessment is viable for neonatal pain assessment in a clinical setting, and the extent of neonatal pain can be evaluated remotely in real-time, which can better identify and treat it and thus improve the neonatal pain condition.

## Figures and Tables

**Table 1:** Patients’ characteristics in this study.

Variables	Total Amount (Proportion, %)
**Gender**	
Female	252 (42.14)
Male	346 (57.86)
**Delivery mode**	
Spontaneous delivery	270 (45.15)
Cesarean section	328 (54.85)
**Pain procedure**	
Arterial blood sampling	81 (13.55)
Heel blood sampling	61 (10.20)
Fingertip blood sampling	85 (14.21)
Intravenous injection	12 (2.01)
Subcutaneous injection	46 (7.69)
Peripheral intravenous cannulation	76 (12.7)
Nasopharyngeal suctioning	65 (10.87)
Retention enema	73 (12.21)
Adhesive removal	68 (11.37)
Wound dressing	31 (5.18)

**Table 2:** ICCs for scoring ratings given by OS-NPA and VB-NPA.

Label Source 1	Label Source 2	ICC	95% CI	*p*-Value
OS-1	VB-1	0.976 (single) 0.988 (averages)	0.972–0.980 0.986–0.990	<0.001 <0.001
OS-2	VB-2	0.983 (single) 0.992 (averages)	0.980–0.986 0.990–0.993	<0.001 <0.001
OS-1	VB-2	0.976 (single) 0.988 (averages)	0.972–0.979 0.986–0.990	<0.001 <0.001
OS-1	OS-2	0.986 (single) 0.993 (averages)	0.984–0.986 0.992–0.994	<0.001 <0.001
OS-2	VB-1	0.976 (single) 0.988 (averages)	0.972–0.979 0.986–0.990	<0.001 <0.001
VB-1	VB-2	0.983 (single) 0.992 (averages)	0.980–0.986 0.990–0.993	<0.001 <0.001

“ICC” stands for intraclass correlation coefficient, “CI” stands for the confidence interval, “OS-NPA” refers to the on-site neonatal pain assessment, and “VB-NPA” refers to the video-based neonatal pain assessment.

**Table 3:** Consistency analysis of NIPS pain grading by OS-NPA and VB-NPA.

		VB-NPA				Kappa Value	*p*-Value
		No Pain	Mild	Moderate	Severe		
**OS-NPA**	**No pain**	97	1	0	0	0.926	<0.001
	**Mild**	2	8	4	1		
	**Moderate**	0	1	32	3		
	**Severe**	0	0	6	443		

The background is highlighted for the diagonal of the confusion matrix.

**Table 4:** Accuracies of different AI methods trained and tested using different label sources.

Label Source	Test	OS-1			OS-2		
Training	Method	[19]	[20]	[21]	[19]	[20]	[21]
**OS-1**	average	0.759	0.783	0.828	0.752	0.779	0.826
	std	0.044	0.040	0.036	0.034	0.031	0.028
**OS-2**	average	0.756	0.769	0.824	0.776	0.786	0.826
	std	0.036	0.039	0.035	0.028	0.024	0.035
**VB-1**	average	0.746	0.769	0.819	0.732	0.754	0.803
	std	0.038	0.038	0.028	0.022	0.023	0.027
**VB-2**	average	0.737	0.747	0.801	0.749	0.761	0.808
	std	0.037	0.042	0.023	0.019	0.031	0.021

‘std’ stands for standard deviation.

## Data Availability

Data related to this study are stored at the Children’s Hospital, the Zhejiang University School of Medicine. Requests for access to the data can be made to the corresponding authors, provided that the necessary ethical clearance has been obtained.

## References

[1] Carbajal R., Rousset A., Danan C., Coquery S., Nolent P., Ducrocq S., Saizou C., Lapillonne A., Granier M., Durand P. (2008). Epidemiology and treatment of painful procedures in neonates in intensive care units. JAMA.

[2] Courtois E., Droutman S., Magny J.F., Merchaoui Z., Durrmeyer X., Roussel C., Biran V., Eleni S., Vottier G., Renolleau S. (2016). Epidemiology and neonatal pain management of heelsticks in intensive care units: EPIPPAIN 2, a prospective observational study. Int. J. Nurs. Stud..

[3] Cruz M.D., Fernandes A.M., Oliveira C.R. (2016). Epidemiology of painful procedures performed in neonates: A systematic review of observational studies. Eur. J. Pain.

[4] Meesters N.J., Simons S.H.P., van Rosmalen J., Holsti L., Reiss I.K.M., van Dijk M. (2019). Acute pain assessment in prematurely born infants below 29 weeks: A long way to go. Clin. J. Pain.

[5] Anand K.J.S. (1998). Clinical importance of pain and stress in preterm neonates. Neonatology.

[6] Slater L., Asmerom Y., Boskovic D.S., Bahjri K., Plank M.S., Angeles K.R., Phillips R., Deming D., Ashwal S., Hougland K. (2012). Procedural pain and oxidative stress in premature neonates. J. Pain.

[7] Walker S.M. (2019). Long-term effects of neonatal pain. Semin. Fetal Neonatal Med..

[8] Perry M., Tan Z., Chen J., Weidig T., Xu W., Cong X.S. (2018). Neonatal pain: Perceptions and current practice. Crit. Care Nurs. Clin..

[9] Sposito N.P.B., Rossato L.M., Bueno M., Kimura A.F., Costa T., Guedes D.M.B. (2017). Assessment and management of pain in newborns hospitalized in a neonatal intensive care unit: A cross-sectional study. Rev. Lat.-Am. Enferm..

[10] Franck L.S., Bruce E. (2009). Putting pain assessment into practice: Why is it so painful?. Pain Res. Manag..

[11] Zamzmi G., Kasturi R., Goldgof D., Zhi R., Ashmeade T., Sun Y. (2017). A review of automated pain assessment in infants: Features, classification tasks, and databases. IEEE Rev. Biomed. Eng..

[12] Merboth M.K., Barnason S. (2000). Managing pain: The fifth vital sign. Nurs. Clin. N. Am..

[13] Grunauer M., Mikesell C., Bustamante G., Cobo G., Sánchez S., Román A.M., Icaza-Freire A.P., Gavilanes A.W.D., Wang N.E., PICU-MIC Research Group (2021). Pain assessment and management in pediatric intensive care units around the world, an international, multicenter study. Front. Pediatr..

[14] Lawrence J., Alcock D., McGrath P., Kay J., MacMurray S.B., Dulberg C. (1993). The development of a tool to assess neonatal pain. Neonatal Netw..

[15] da Motta G.d.C.P., Schardosim J.M., da Cunha M.L.C. (2015). Neonatal infant pain scale: Cross-cultural adaptation and validation in Brazil. J. Pain Symptom Manag..

[16] Napiórkowska-Orkisz M., Gutysz-Wojnicka A., Tanajewska M., Sadowska-Krawczenko I. (2022). Evaluation of methods to minimize pain in newborns during capillary blood sampling for screening: A randomized clinical trial. Int. J. Environ. Res. Public Health.

[17] Suraseranivongse S., Kaosaard R., Intakong P., Pornsiriprasert S., Karnchana Y., Kaopinpruck J., Sangjeen K. (2006). A comparison of postoperative pain scales in neonates. Br. J. Anaesth..

[18] Yao W.Y., Petrini M., Deng W.L., Wu H.L., Tu H.X. (2011). Effects of glucose administering approaches on reducing neonatal pain during heel lance procedures. Chin. J. Nurs..

[19] Zamzmi G., Pai C.-Y., Goldgof D., Kasturi R., Ashmeade T., Sun Y. (2019). A comprehensive and context-sensitive neonatal pain assessment using computer vision. IEEE Trans. Affect. Comput..

[20] Min L., Sun Y., de With P.H.N. (2021). Video-based infant monitoring using a CNN-LSTM scheme. Proc. SPIE.

[21] Salekin M.S., Zamzmi G., Goldgof D., Kasturi R., Ho T., Sun Y. (2021). Multimodal spatio-temporal deep learning approach for neonatal postoperative pain assessment. Comput. Biol. Med..

[22] Dagnaes-Hansen J., Mahmood O., Bube S., Bjerrum F., Subhi Y., Rohrsted M., Konge L. (2018). Direct observation vs. video-based assessment in flexible cystoscopy. J. Surg. Educ..

[23] Salekin M.S., Mouton P.R., Zamzmi G., Patel R., Goldgof D., Kneusel M., Elkins S.L., Murray E., Coughlin M.E., Maguire D. (2021). Future roles of artificial intelligence in early pain management of newborns. Paediatr. Neonatal Pain.

[24] Cheng X., Zhu H., Mei L., Luo F., Chen X., Zhao Y., Chen S., Pan Y. (2022). Artificial intelligence based pain assessment technology in clinical application of real-world neonatal blood sampling. Diagnostics.

[25] Scaffidi M.A., Grover S.C., Carnahan H., Yu J.J., Yong E., Nguyen G.C., Ling S.C., Khanna N., Walsh C.M. (2018). A prospective comparison of live and video-based assessments of colonoscopy performance. Gastrointest. Endosc..

[26] Sun Y., Kommers D., Wang W., Joshi R., Shan C., Tan T., Aarts R.M., van Pul C., Andriessen P., de With P.H.N. Automatic and continuous discomfort detection for premature infants in a NICU using video-based motion analysis. Proceedings of the 2019 41st Annual International Conference of the IEEE Engineering in Medicine and Biology Society (EMBC).

[27] Hoti K., Chivers P.T., Hughes J.D. (2021). Assessing procedural pain in infants: A feasibility study evaluating a point-of-care mobile solution based on automated facial analysis. Lancet Digit. Health.

[28] Branco A., Fekete S.M.W., Rugolo L.M.S.S., Rehder M.I. (2007). The newborn pain cry: Descriptive acoustic spectrographic analysis. Int. J. Pediatr. Otorhinolaryngol..

[29] Almeida M., Zhuang Y., Ding W., Crouter S.E., Chen P. (2021). Mitigating class-boundary label uncertainty to reduce both model bias and variance. ACM Trans. Knowl. Discov. Data.

